# The Concentration of Soluble Extracellular Amyloid-β Protein in Acute Brain Slices from CRND8 Mice

**DOI:** 10.1371/journal.pone.0015709

**Published:** 2010-12-29

**Authors:** Jack Waters

**Affiliations:** Department of Physiology, Feinberg School of Medicine, Northwestern University, Chicago, Illinois, United States of America; Case Western Reserve University, United States of America

## Abstract

**Background:**

Many recent studies of the effects of amyloid-β protein (Aβ) on brain tissue from amyloid precursor protein (APP) overexpressing mice have concluded that Aβ oligomers in the extracellular space can profoundly affect synaptic structure and function. As soluble proteins, oliomers of Aβ can diffuse through brain tissue and can presumably exit acute slices, but the rate of loss of Aβ species by diffusion from brain slices and the resulting reduced concentrations of Aβ species in brain slices are unknown.

**Methodology/Principal Findings:**

Here I combine measurements of Aβ_1–42_ diffusion and release from acute slices and simple numerical models to measure the concentration of Aβ_1–42_ in intact mice (*in vivo*) and in acute slices from CRND8 mice. The *in vivo* concentration of diffusible Aβ_1–42_ in CRND8 mice was 250 pM at 6 months of age and 425 pM at 12 months of age. The concentration of Aβ_1–42_ declined rapidly after slice preparation, reaching a steady-state concentration within one hour. 50 µm from the surface of an acute slice the steady-state concentration of Aβ was 15–30% of the concentration in intact mice. In more superficial regions of the slice, where synaptic physiology is generally studied, the remaining Aβ is less than 15%. Hence the concentration of Aβ_1–42_ in acute slices from CRND8 mice is less than 150 pM.

**Conclusions/Significance:**

Aβ affects synaptic plasticity in the picomolar concentration range. Some of the effects of Aβ may therefore be lost or altered after slice preparation, as the extracellular Aβ concentration declines from the high picomolar to the low picomolar range. Hence loss of Aβ by diffusion may complicate interpretation of the effects of Aβ in experiments on acute slices from APP overexpressing mice.

## Introduction

Amyloid-β protein (Aβ) molecules aggregate in solution, forming soluble oligomers and insoluble aggregates [1]–[3]. The latter are a major component of senile plaques, a hallmark of Alzheimer's disease (AD). The discovery that neurodegeneration and cognitive impairment correlate only weakly with plaque count and more strongly with soluble or total Aβ load has led to renewed appreciation of the deleterious effects of soluble forms of Aβ [4]–[11].

The effects of soluble Aβ include impaired synaptic transmission, typically observed in mouse models in which point mutations in the amyloid precursor protein (APP) or other AD-linked genes lead to APP overexpression and Aβ accumulation [12]–[21]. In these mouse models, neurons and synapses are exposed to elevated concentrations of Aβ for months, but soluble Aβ can also act rapidly. For example, exposure to soluble Aβ for a few minutes impairs hippocampal synaptic plasticity [18], [19], [22]–[24]. Hence both prolonged and rapid elevation of the extracellular concentration of soluble Aβ species can alter neuronal function.

Much of our understanding of the effects of Aβ on cellular and synaptic function originates from experiments on acute brain slices, prepared from APP overexpressing mice. Acute slices are often used to study synaptic physiology as cells and synapses are accessible and many short-range synaptic connections remain intact. One potential problem is the loss of Aβ after slice preparation. Soluble Aβ species are mobile in solution and extracellular soluble Aβ may exit slices by diffusion. Hence soluble Aβ is presumably lost from slices and the steady-state concentration of extracellular Aβ in an acute slice is likely to be lower than that in the brains of intact mice (*in vivo*). However, neither the steady-state concentrations of Aβ monomers and oligomers nor the kinetics of the decline in extracellular Aβ concentration have been determined in acute slices from APP overexpressing mice.

Here I use simple numerical models, fluorescence microscopy and enzyme-linked immunosorbent assays (ELISAs) to determine the extracellular concentration of soluble Aβ_1–42_ in acute slices from CRND8 mice. I find that the extracellular Aβ_1–42_ concentration is elevated after slice preparation, but rapidly declines below the initial concentration, reaching steady-state within ∼30–60 minutes. Hence in slice physiology experiments, in which measurements are typically made 30 minutes to several hours after slice preparation, the extracellular concentration of soluble Aβ_1–42_ is a small fraction of the *in vivo* concentration, raising the possibility that some of the effects of extracellular soluble Aβ species are absent or attenuated.

## Methods

### Ethics statement

All experiments and procedures involving animals were approved by the Northwestern University Institutional Animal Care and Use Committee (IACUC).

### Rate of diffusion of Aβ in brain tissue

The rate of diffusion of a particle in solution is determined by its diffusion coefficient (D). The diffusion coefficient for amyloid-β (Aβ) monomer in aqueous solution at 25°C has been estimated from NMR measurements as between 1.4×10^−6^ and 2.1×10^−6^ cm^2^/s [25], [26]. The diffusion coefficient is linearly dependent on absolute temperature and is therefore approximately 4% greater at 37°C than at 25°C. Hence the literature indicates that the diffusion coefficient for Aβ monomer in aqueous solution at 37°C is approximately 1.8×10^−6^ cm^2^/s and this is the value used here.

Diffusion in brain tissue is impeded by a variety of barriers, including cells and extracellular matrix. The effective diffusion coefficient (D_eff_) in brain tissue is related to the diffusion coefficient for free diffusion by:
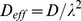
(1)where λ is the tortuosity factor.

For brain tissue, λ is ∼1.7 [27], [28]. Hence D_eff_ = 0.623×10^−6^ cm^2^/s for Aβ monomer in brain tissue at 37°C.

The distribution of Aβ after release from a point source may be calculated using the following equation [27], [29]:
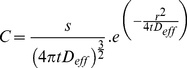
(2)where C is the concentration of the diffusing species

S is the amount of the diffusing species

D_eff_ is the effective diffusion coefficient

t is time

r is the distance (radius) of diffusion in three dimensions

### Random walk model of Aβ diffusion

**Figure 1:**
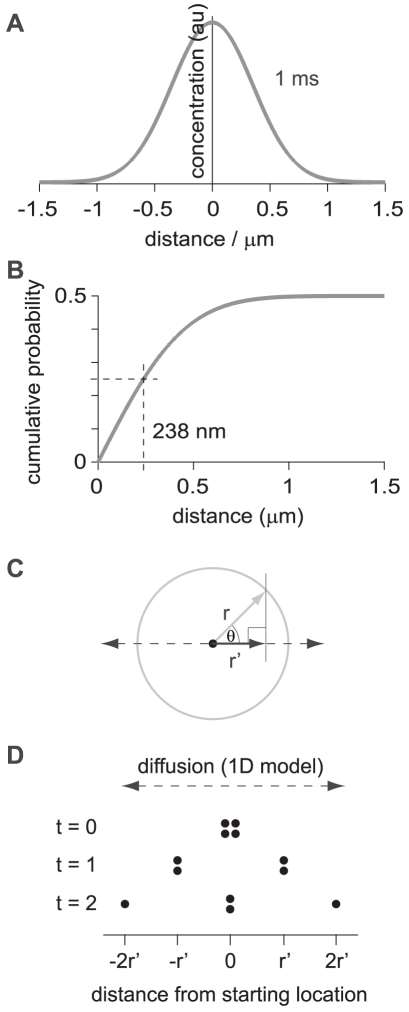
Random-walk model of Aβ diffusion in brain tissue. (**A**) Distribution of Aβ monomer 1ms after release from a point source (at distance = 0). The concentration of Aβ is given in arbitrary units (au). Curve calculated using equation 2, with D_eff_ = 0.623×10^−6^ cm^2^/s. (**B**) Cumulative distribution of Aβ monomer 1ms after release from a point source (at distance = 0), derived by integrating the curve in panel A from zero to infinity. Half the diffusing molecules (cumulative probability 0.25) are within 238 nm of the point source. (**C**) Calculation of mean diffusion distance in one dimension from that in three dimensions. A particle is released from a point source. In time = t, it diffuses a mean distance of r (black arrow) in a random angle (θ) to the axis of diffusion (dashed line). The mean distance of diffusion in one dimension (r′; black arrow) occurs when θ = 45°. (**D**) Diffusion of four molecules through two iterative steps of the random walk model. At each step half of the molecules in each location move r′ towards infinity and half towards negative infinity.

**Figure 2:**
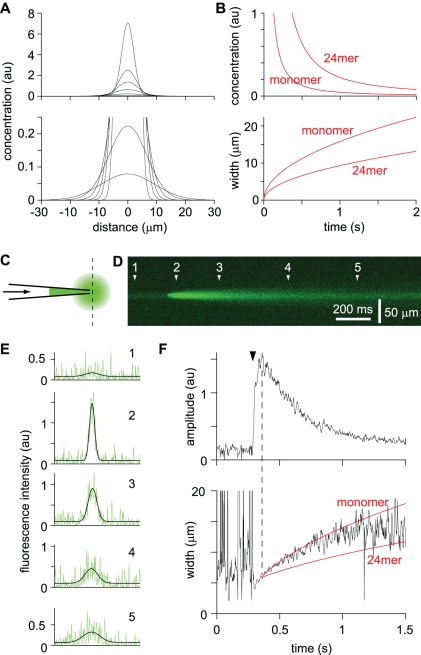
Diffusion of fluorescent Aβ_1–42_ in brain tissue. (**A**) Expected Gaussian distributions of Aβ monomer after release from a point source, calculated using an effective diffusion coefficient of 0.623×10^−6^ cm^2^/s for monomer and 0.216×10^−6^ cm^2^/s for 24mer. Distributions are shown (on two different scales) for 100, 200, 300, 500, 1000 and 2000 ms after release from a point source. Concentrations are given in arbitrary units (au). (**B**) Plots showing the decrease in peak amplitude and increase in width of the Gaussian distributions during the first two seconds after release from a point source, calculated for monomer and 24mer. (**C**) Schematic showing the orientation of the 2-photon line scan with respect to the pipette containing fluorescent Aβ_1–42_ (green fill). Fluorescence was measured repeatedly by scanning the laser excitation repeatedly along a line (line scan) oriented perpendicular to the long axis of the pipette. Fluorescent Aβ_1–42_ was ejected into the tissue (green circle) by the application of pressure to the rear of the pipette (arrow). (**D**) Result of one trial, showing the increase in fluorescence intensity following pressure ejection of fluorescent Aβ_1–42_. Distance along the scanned line is shown on the vertical axis and time on the horizontal axis. The distributions of fluorescence along the line is shown in panel E for the 5 marked points in time (arrow heads). (**E**) Fluorescence intensities (green) and Gaussian fits (black) for the five time points indicated in panel F. Fluorescence intensity is displayed in arbitrary units (au). (**F**) The amplitude and width of the Gaussian fits, plot as a function of time, for the trial shown in panel D. Fluorescent Aβ_1–42_ was ejected from the pipette with a single 20 ms pulse of pressure at the arrow head. The fluorescence amplitude peaked several milliseconds later (dashed line). The widths of the Gaussian fits were variable before pressure ejection because the fits to the low background fluorescence (probably autofluorescence of the slice and leakage of fluorescence from the pipette tip) were variable. During and after pressure ejection the width became more consistent, indicating reliable Gaussian fits, as seen in panel E. The width of the Gaussian fits increased over time. For comparison, the expected rates of expansion of the widths of the Gaussian fits, starting from the time at which the fluorescence intensity peaked, were calculated for monomer and 24mer and are overlaid in red.

The distribution of Aβ monomer (calculated using equation 2) as a function of distance from a point source (distance = 0) after 1ms of diffusion is shown in figure 1A. Integration of this curve from the point of release to infinity gives the cumulative probability of finding a single molecule of Aβ with increasing distance from the point of release (figure 2B). (Note that the cumulative probability approaches 0.5 as distance tends towards infinity. This is because half of the particles diffuse towards infinity and half in the opposite direction, towards minus infinity.)

From the cumulative probability plot shown in figure 2B I derived the median radius of diffusion (r). The median radius is the distance traveled by half the molecules, i.e. cumulative probability = 0.25. For Aβ monomer in brain tissue, the median radius of diffusion in 1 ms is 238nm. Hence half of the diffusing Aβ molecules are found between +238 and −238 nm of the point source 1ms after their release.

In the model Aβ diffuses from brain tissue into ACSF or a layer of agarose. Tissue, ACSF and agarose are homogenous media separated by planar boundaries that are parallel to each other and the concentrations of Aβ are uniform across each plane parallel to these boundaries (and perpendicular to the axis of diffusion). This greatly simplifies the model since Aβ that moves laterally away from the axis of diffusion will, on average, be replaced by an equal amount of Aβ moving towards the axis of diffusion. Hence diffusion can be described using a relatively simple one-dimensional model with the axis of diffusion perpendicular to the boundaries between tissue, ACSF and agarose. However, the radius of diffusion calculated from equation 2 is for diffusion in three dimensions. I therefore converted the radius of diffusion (in 3 dimensions) to an equivalent distance of diffusion in one dimension, along the axis (r′). This is illustrated in figure 2C. In time = t, a particle diffuses distance r (grey arrow) in a random direction, i.e. at a random angle θ with respect to the axis of diffusion in one dimension (dashed line in figure 2C). Hence after time = t, the particle is at a random location on the surface of a sphere of radius r (drawn as a circle in figure 2C). On average the particle travels at 45 degrees to the axis of diffusion in one dimension (there being an equal number of angles between θ and zero and θ and 90 degrees and, therefore and equal probability of θ being less and greater than 45 degrees). The mean distance traveled along the axis of diffusion in one dimension (r′) will therefore be
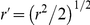
Hence the mean distance of one-dimensional diffusion of Aβ in 1 ms (for which r = 238 nm) is 168 nm.

One-dimensional random-walk models were constructed to quantify diffusion. Diffusion was simulated as a series of stepwise displacements of Aβ, where in each time-step half of the material moved towards infinity and the other half towards negative infinity. This is illustrated in figure 2D in which the position of 4 molecules are shown at three subsequent time points. Calculations were performed with 5–10 nm spatial resolution and 1 ms to 1 s time steps.

Aβ multimers were assumed to diffuse as globular proteins, for which
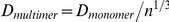
where n is the number of monomers that aggregate to form the multimer.

Hence for an oligomer composed of 24 monomers (24mer), D = 0.624×10^−6^ cm^2^/s and D_eff_ = 0.216×10^−6^ cm^2^/s.

Constant turn-over of Aβ was included in some model calculations by adjusting the concentrations of Aβ at every location within the tissue after each iterative step of the model. Monomer and oligomers were all assumed to turn-over at a constant rate of 0.015% per second (unless otherwise stated) which corresponds to a half-life of 1.28 hours. Published measurements for the half-life of Aβ are 0.7 to 3.8 hours in mice [30], [31] and ∼8–9 hours in human CNS [32].

Several similar models were employed to calculate Aβ concentrations under slightly different conditions. Model #1 simulated a brain slice bathed in a practically infinite volume of circulating ACSF, such as in a large-volume holding chamber. After each iterative step of this model, Aβ that had exited the slice into the ACSF was eliminated from the model. Diffusion of Aβ from the brain in an intact mouse was simulated in model #2, in which the brain surface was covered with a layer of agarose. Aβ exiting the brain entered the agarose. Diffusion through the agarose was modeled as diffusion in free solution, using a diffusion coefficient of 1.8×10^−6^ cm^2^/s for Aβ monomer and 0.624×10^−6^ cm^2^/s for 24mer. A half-life for Aβ of 1.28 hours was employed in model #2. Model #3 simulated a variable ‘recovery’ period and subsequent ‘measurement’ period. The tissue concentration of Aβ was first calculated (using model #1) during a recovery period in which the slice was in a practically infinite volume of circulating ACSF. The slice was then transferred to a smaller-volume chamber containing 250 µl ACSF, in which Aβ which exits the slice accumulates in the ACSF. The ACSF was assumed to be well-mixed and after each iterative step Aβ that had exited the slice into the ACSF was therefore redistributed evenly throughout the ACSF. Model #4 simulated a slice placed in a small-volume chamber containing 250 µl ACSF immediately after preparation. The ACSF was assumed to be well-mixed and after each iterative step Aβ that had exited the slice into the ACSF was therefore redistributed evenly throughout the ACSF. Model #5 simulated exogenous application of Aβ to a slice. The initial concentration of Aβ in the slice was zero. The slice was bathed in an infinite volume of Aβ-containing ACSF and after each iterative step, the Aβ concentration in the ACSF was restored to 1. Turn-over of Aβ was excluded from this model.

Simplifying assumptions were used to calculate the rates of diffusion across boundaries between media in which the diffusion coefficients for Aβ were different. In slice experiments the ACSF was constantly stirred. In the corresponding models (#3 and #4) I therefore assumed that the ACSF contained a homogenous concentration of Aβ. Hence at the end of each iteration of the model, during which Aβ would have been exchanged between tissue and ACSF using the effective diffusion coefficient for the tissue, the concentration of Aβ at all locations in the ACSF was set to the mean ACSF Aβ concentration. In the *in vivo* model (#2), it was necessary to simulate diffusion through across a tissue-agarose boundary and through the agarose. After each iteration, the concentration across all locations within one diffusion coefficient of the boundary was averaged. To prevent any Aβ crossing the agarose-coverslip boundary, particles less than one diffusion coefficient from the boundary were permitted to move only away from the coverslip, 50% of the particles remaining stationary in each step.

All simulations were implemented in Igor Pro 6.0 (Wavemetrics, Eugene, OR) using custom-written routines. The code for model #1 is available as supporting information (Code S1).

### Diffusion of fluorescent Aβ_1–42_ in acute slices

Diffusion of fluorescently labeled Aβ_1–42_ (HiLyte Fluor 488 labeled Aβ(1–42), Anaspec, Fremont, CA) was modeled using the diffusion coefficients for unlabeled Aβ_1–42_. This is an approximation since the fluorescent tag increases the relative molecular mass of the molecule from 4514.1 to 4870.5, which presumably results in a small reduction in the diffusion coefficient. When modeling diffusion of fluorescent Aβ_1–42_ I also assumed that there was no turn-over of this molecule in brain tissue.

I measured diffusion of fluorescent Aβ_1–42_ in acute slices. An adult wild-type mouse was deeply anesthetized with isoflurane and decapitated and the brain was rapidly removed into cold artificial cerebrospinal fluid (ACSF). ACSF composition was (in mM): 125 NaCl, 2.5 KCl, 1.25 NaH_2_PO_4_, 20 NaHCO_3_, 5 HEPES, 25 Glucose, 2 CaCl_2_, 1MgCl_2_, pH 7.3, oxygenated with 95% O_2_/5% CO_2_. Parasagittal slices 200 µm thick were prepared using a vibrating slicer (Vibratome, St. Louis, MO) and maintained in the above solution at room temperature for 2 hours before transfer to the microscope stage. On the microscope stage the slice was constantly perfused with the above solution at 35–37°C.

Fluorescence was measured by 2-photon fluorescence microscopy using a custom built microscope based on an Olympus BX51 frame. The specimen was illuminated with 840 nm light from a Ti:sapphire laser (Chameleon Ultra, Coherent, 80 MHz repetition rate; 100–150 fs pulse width). Excitation light was focused onto the specimen using a ×40, NA 0.8 or ×20, NA 1.0 water-immersion objective (Olympus, Center Valley, PA). Emitted light was collected in the epifluorescence configuration through a 680 nm dichroic reflector and an infrared-blocking emission filter (ET700sp-2p, Chroma Technology, Bellows Falls, VT). Fluorescence was detected via a 490–560 bandpass filter (Chroma Technology) using a photomultiplier tube (R6357, Hamamatsu). Scanning and image acquisition were controlled using custom software written in Labview (National Instruments, Austin, TX).

The rate of diffusion of fluorescent Aβ_1–42_ was measured in acute slices by 2-photon fluorescence microscopy. Fluorescent Aβ_1–42_ was applied locally by pressure ejection from a pipette. HiLyte Fluor 488 labeled Aβ(1–42) was dissolved at 100 µM in modified ACSF (in mM): 135 NaCl, 5.4 KCl, 1 MgCl2, 1.8 CaCl2, 5 HEPES, pH 7.3. A pipette with a tip diameter of ∼1 µm was filled with 100 µM fluorescent Aβ_1–42_ and the tip was positioned in stratum radiatum of the hippocampus, ∼100 µm below the surface of the slice. Fluorescent Aβ_1–42_ was ejected from the pipette with brief a pulse of positive pressure (10–50 ms, 20–30 psi) controlled by a pressure ejector (Toohey Spritzer, Fairfield, NJ). Fluorescence was measured a few µm from the pipette tip using line scans, in which the tissue near the tip of the pipette was scanned repeatedly at 2ms intervals by the excitation laser.

I also measured diffusive loss of fluorescent Aβ_1–42_ from acute slices. Slices were prepared as described above and immediately placed into 50 µM fluorescently labeled Aβ_1–42_ in modified ACSF(in mM): 135 NaCl, 5.4 KCl, 1 MgCl2, 1.8 CaCl2, 5 HEPES, pH 7.3, oxygenated with 95% O_2_/5% CO_2_. Solution was constantly stirred by placing the plate on an orbital shaker. After 2 hours (to allow the concentration of fluorescent Aβ_1–42_ throughout the slice to approach 50 µM), the slice was transferred to the stage of the 2-photon microscope. The fluorescence within the slice was imaged at regular intervals during perfusion with ACSF (no Aβ or fluorophore) warmed to 35–37°C.

Measuring loss of fluorescent Aβ_1–42_ required repeated imaging in the single location for up to ∼1 hour. Hence photobleaching and drift in x-, y- and z-axes could potentially confound the results. To minimize photobleaching I used a relatively high concentration of Aβ_1–42_, which was bright enough to enable the use of low intensity laser excitation. In addition after imaging the decline in fluorescence through time I verified that the intensity in the imaged region was similar to that in neighbouring regions of the slice. The presence of identifiable structures in the image throughout the imaging period suggested that drift was negligible, particularly parallel to the optical axis of the microscope (x- and y-axes). To further exclude the possibility of drift perpendicular to the optical axis (z-axis) I checked that the slice surface was still 100 µm from the imaging site after measurements of fluorescence through time were complete. Through these controls I ensured that neither photobleaching nor drift in the relative position of the slice and focal position effected these measurements significantly.

### Measurement of Aβ_1–42_ release from acute slices

Coronal slices 300 µm thick were prepared from CRND8 mice as described above. To measure release of Aβ species, each slice was transferred to one well of a 24-well plate immediately after preparation. The well contained 250 µl of modified ACSF (in mM): 135 NaCl, 5.4 KCl, 1 MgCl2, 1.8 CaCl2, 5 HEPES, pH 7.3, oxygenated with 95% O_2_/5% CO_2_. Solution was constantly stirred by placing the plate on an orbital shaker. Duplicate measurements of Aβ_1–42_ concentration in the ACSF were made using a sandwich enzyme-linked immunosorbent assay (ELISA) kit (high sensitive human beta amyloid(1–42) ELISA kit, Wako Chemicals, Richmond VA), which measures Aβ_1–42_ concentrations in the 1–20 pM range. To correct for differences in the volumes of individual slices, we measured the wet weight of each slice at the end of the experiment and normalized Aβ_1–42_ release to that of a 25 mg slice.

Hence Aβ_1–42_ concentration was measured after release into a relatively small volume of ACSF. 250 µl was selected as the volume of ACSF as this was the smallest volume compatible with duplicate measurements using the ELISA kit, which requires 100 µl per measurement. Hence the volume of a 25 mg slice was 10% of the volume of ACSF. Minimizing the volume of ACSF was necessary as the total amount of Aβ_1–42_ released from a brain slice is likely to be small.

In some experiments slices were pre-washed in ACSF before the measurement of Aβ_1–42_ release into ACSF. The pre-wash consisted of incubating the slices in a large volume (∼250 ml) of constantly-circulating ACSF at 35°C.

Totals numbers of mice used in these experiments, by age range, were: two P96–114; six P127–144, three P152–163; two 203–233.

### Measurement of Aβ release in vivo

To measure the extracellular diffusible Aβ_1–42_ concentration *in vivo* (i.e. in intact animals) I implanted a small chamber over frontal cortex, placed a small volume of agarose in the chamber and in contact with the brain for 48 hours and later assayed the concentration of Aβ_1–42_ in the agarose.

A mouse was deeply anesthetized with isoflurane (2–2.5%, inhaled) such that pinch-withdrawl and palpebral reflexes were eliminated. The skin was reflected and a small aluminium plate was attached to the skull with dental acrylic. A large craniotomy (diameter ∼5–6 mm) was opened over frontal cortex, centered ∼1 mm posterior to bregma. The brain was covered with 40–60 mg of 0.75–1% agarose (w/v) in ACSF. The craniotomy and agarose were covered with an 8 mm-diameter coverslip and sealed using dental acrylic. The mouse was allowed to recover and was returned to its home cage.

The model predicts that the concentrations of diffusible extracellular Aβ species in the agarose are close to the concentrations in brain tissue after ∼30 hours and the mouse was therefore left in its home cage for ∼60 hours. It was then re-anesthetized. The agarose was collected and placed in 350 µl of ACSF for ≥4 hours, which is sufficient for the Aβ_1–42_ to diffuse into the ACSF and reach an equilibrium concentration. The ACSF was then collected, diluted (to bring the final ACSF Aβ_1–42_ concentration into the appropriate range for the ELISA kit) and its Aβ_1–42_ content was determined with the ELISA kit. The agarose was subsequently weighed to determine the dilution factor into ACSF and thereby calculate the concentration of Aβ_1–42_ in brain tissue.

A total of 9 mice were used for *in vivo* experiments, with ages from P100 to P370.

## Results

### Rate of diffusion of Aβ_1–42_ in brain tissue

To model the diffusion of Aβ out of a brain slice, we need to know the effective diffusion coefficient of Aβ in brain tissue. The diffusion coefficient for Aβ_1–42_ monomer in aqueous solution is available from the published literature and from this value the effective diffusion coefficient of Aβ_1–42_ monomer in brain tissue was estimated at 0.623×10^−6^ cm^2^/s (see Methods section). Soluble forms of Aβ include monomers and oligomers of up to 24 monomers [1] (24mers). Hence I also considered diffusion of 24mers of Aβ_1–42_ with an effective diffusion coefficient in brain tissue of 0.216×10^−6^ cm^2^/s (see Methods section). To verify that these estimates of effective diffusion coefficients accurately represent diffusion of Aβ_1–42_ in the brain, I measured the diffusion of fluorescently-tagged Aβ_1–42_ in hippocampal tissue and compared the spread of fluorescent Aβ_1–42_ with the calculated distribution expected from theory.

I first calculated the expected distribution of Aβ_1–42_ in brain tissue after release from a point source. This distribution is Gaussian, with the concentration declining from a peak at the site of release. As molecules diffuse away from the source of release over time, the shape of the distribution remains Gaussian, with the peak amplitude declining and the width increasing with time (figure 2A and 2B). The amplitude and width of the Gaussian distribution at any moment in time will depend on the diffusion coefficient of the diffusing species. I therefore calculated the expected distributions for both monomer and 24mer using their respective diffusion coefficients.

I then measured the rate of diffusion of fluorescent Aβ_1–42_ in the acute hippocampal slice. A pipette with a tip diameter of ∼1 µM was filled with 100 µM fluorescent Aβ_1–42_ (in ACSF) and was inserted into stratum radiatum, such that the tip was ∼100 µm below the surface of the slice. A small volume of fluorescently-tagged Aβ_1–42_ was ejected into the slice by the brief application of pressure (10–50 ms, 20–30 psi). The diffusion of Aβ_1–42_ through the tissue near the tip of the pipette was measured by 2-photon fluorescence microscopy (figure 2C and 2D).

During pressure ejection the fluorescence intensity near the tip of the pipette increased rapidly, reaching a peak a few milliseconds after ejection ended (figure 2D–F). As expected, the fluorescence intensity was well-described by a Gaussian distribution with the peak of the fluorescence close to the tip of the pipette. Furthermore the peak amplitude declined and the width increased with time (figure 2D–F). Hence pressure ejection of Aβ_1–42_ approximates release of Aβ_1–42_ from a point source. Importantly, the rate at which the width of the Gaussian fit increased was similar to that expected from theoretical calculations (figure 2F). I measured diffusion in 6 locations, following Aβ_1–42_ diffusion for 705±145 ms, during which time the width of the Gaussian fit increased from 6.5±1.0 µm to 13.7±2.46 µm. By comparison, expansion of the Gaussian distribution from 6.5 to 13.7 µm was expected, from theoretical calculations, to take 580 ms for monomer and 1.68 seconds for 24 mer. Hence these measurements confirm that Aβ_1–42_ diffuses rapidly through the extracellular space in brain tissue and that this diffusion can be accurately modeled using effective diffusion coefficients of 0.623×10^−6^ cm^2^/s for monomers and 0.216×10^−6^ cm^2^/s for 24mers of Aβ_1–42_.

### Model predicts substantial loss of extracellular diffusible Aβ species from acute brain slices within 1 hour

**Figure 3:**
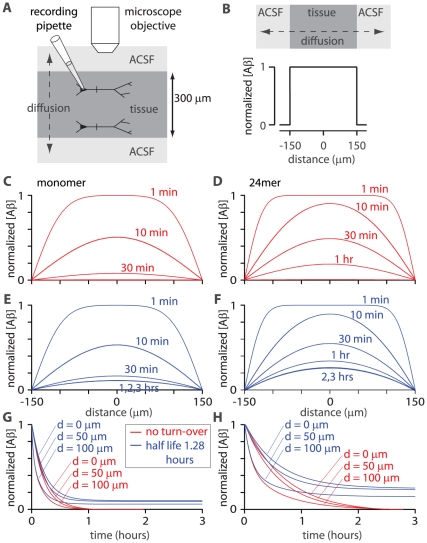
Model predicts rapid loss of diffusible Aβ from acute brain slices. (**A**) Schematic illustration of model #1, in which a 300 µm-thick tissue slice is bathed in a large volume of ACSF, as commonly employed in slice physiology and imaging experiments. We calculated diffusion in the vertical axis (dashed line). (**B**) Initial concentration of Aβ as a function of location. Tissue-ACSF boundaries are at distances of +150 and −150 µm. Initial concentration in the tissue is 1, with the result that all concentrations in the model are normalized to the initial concentration in brain tissue. (**C and D**) Concentrations of monomer (C) and 24mer (D) at all locations in the brain slice (plot as a function of distance from the center of the brain slice) at 1 minute, 10 minutes, 30 minutes, 1 hour, 2 hours and 3 hours in the absence of Aβ turn-over. (**E and F**) Concentrations of monomer (E) and 24mer (F) at all locations in the brain slice (plot as a function of distance from the center of the brain slice) at 1 minute, 10 minutes, 30 minutes, 1 hour, 2 hours and 3 hours with a half-life for Aβ of 1.28 hours. (**G and H**) Concentrations of monomer (G) and 24mer (H) as a function of time at three different locations within the brain slice. Distances (0, 50, 100 µm) are with respect to the center of the slice. Red lines: no turn-over of Aβ; blue lines: half-life of 1.28 hours.

Using these diffusion coefficients, I calculated the expected rate of loss of diffusible Aβ species from the extracellular space of an acute slice, using a simple random walk model of diffusion. The aim was to model loss of Aβ in slice physiology experiments, such as experiments in which many investigators have studied the effects of Aβ species on synaptic transmission. In such experiments, the brain is typically cut into slices that are 200- to 400 µm-thick and these slices are then incubated in a large volume of ACSF for 30 minutes to several hours before electrophysiological recordings are obtained. I therefore modeled diffusion from a 300 µm-thick section of tissue, bathed on both sides by ACSF (figure 3A). Aβ which exited the tissue into the ACSF was instantly removed. The initial concentrations of Aβ were one in tissue and zero in ACSF (figure 3B), with the result that all concentrations in the model are normalized to the initial concentration of Aβ in the extracellular space. Using this approach I calculated the concentrations of monomer and 24mer throughout the slice (figure 3C–H).

Aβ concentrations were first calculated for the simplest scenario, in which Aβ is not gained or lost (other than by diffusion) from the tissue (no turn-over; figure 3C, D). However, both Aβ_1–42_ and Aβ_1–40_ have relatively short half-lives in the CNS (0.7 to 4 hours in APP overexpressing mice [30], [31]). Hence Aβ concentrations were also calculated with constant turn-over of Aβ, assuming a half-life of Aβ of 1.28 hours (figure 3E,F). In both scenarios, the concentrations of monomer and 24mer were greatly reduced at the edges of the slice within a minute of slice preparation (figure 3C–F). Even deep in the slice, the concentrations of monomer and 24mer were dramatically reduced. Monomer reached a steady-state concentration throughout the slice in 30–60 minutes and 24mer after 1–2 hours (figure 3G,H). Hence the model predicts that the diffusive loss of Aβ from the extracellular space in an acute slice is rapid and profound.

### Loss of fluorescently-tagged Aβ_1–42_ from acute brain slices

**Figure 4:**
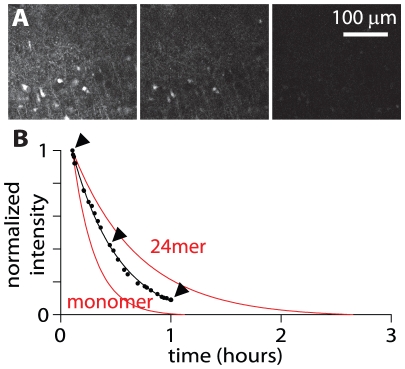
Loss of fluorescent Aβ_1–42_ from acute slices. (**A**) Three 2-photon fluorescence images acquired from a focal plane 100 µm below the surface of an acute slice. The slice was pre-incubated with 50 µM fluorescent Aβ_1–42_ in ACSF for 3.5 hours and a series of images were acquired over the subsequent hour, during perfusion of the slice with Aβ-free ACSF. From left to right, these images were acquired after 6.37, 26.7 and 60.1 minutes of perfusion with Aβ-free ACSF. (**B**) Time course of the decline in fluorescence with time in Aβ-free ACSF for the site shown in panel A. Each point represents intensity in one image. The decay in fluorescence was fit with an exponential with a time constant of 25.2 minutes (black line). The expected rates of loss of monomer and 24mer (normalized to the initial intensity in the first image), calculated using the diffusion model (model #1; figure 3), are shown in red. Arrow heads indicate the three images in panel A.

To confirm that soluble Aβ is indeed rapidly lost from the extracellular space in acute slices I loaded acute slices with fluorescent Aβ_1–42_ and monitored the decline in intensity as fluorescent Aβ_1–42_ diffused out of the slice. Immediately after preparation acute slices were placed in ACSF containing 50 µM fluorescent Aβ_1–42_. After 2–3.5 hours (long enough for the concentration of fluorescent Aβ_1–42_ in the extracellular space to approach 50 µM; see ‘Kinetics of exogenous Aβ accumulation in slices’ section, below) the slice was placed on the stage of the 2-photon microscope and the fluorescence intensity 100 µM below the upper surface of the slice was monitored for up to ∼1 hour as the slice was perfused with fresh ACSF, containing no Aβ or fluorophore. As expected the fluorescence intensity declined with time (n = 3 slices; figure 4). Furthermore, the decline in intensity with time matched the rate of loss of Aβ expected from the model (figure 4B), occurring with a time constant of 18.0±7.0 minutes (n = 3 slices). I conclude that extracellular Aβ is lost rapidly from acute slices with the time course predicted by the diffusion model.

### The concentration of diffusible extracellular Aβ_1–42_ in CRND8 mice *in vivo*

Hence it seems likely that endogenous soluble Aβ species are rapidly lost from the extracellular space in acute slices with the time course predicted by the model, but the diffusion of fluorescent Aβ_1–42_ might differ from that of endogenous Aβ_1–42_. For example, the fluorescence tag might affect diffusion or the high concentration of fluorescent Aβ_1–42_ that was, necessarily, added to the slice might stimulate or saturate pathways of Aβ metabolism or clearance. I therefore sought to determine the rate of loss of Aβ_1–42_ from acute slices from CRND8 mice, which overexpress APP and accumulate Aβ_1–42_ in the hippocampus and neocortex. The concentration of endogenous Aβ_1–42_ in the extracellular space of an acute slice is difficult or perhaps impossible to measure directly. My aim was therefore to combine the model with measurements of Aβ_1–42_ release from acute slices to determine what proportion of diffusible Aβ remains in the extracellular space as a function of time after slice preparation.

First I measured the concentration of diffusible, extracellular Aβ_1–42_ in the intact brain. A craniotomy was cut over frontal cortex and a small reservoir of agarose was placed in contact with the brain surface. In this preparation Aβ species diffuse from the brain and into the agarose and the concentrations of the mobile Aβ species in the agarose increase until their concentrations equal those in the brain. The agarose was then removed and the concentration of Aβ_1–42_ in the agarose was measured with an ELISA kit.

**Figure 5:**
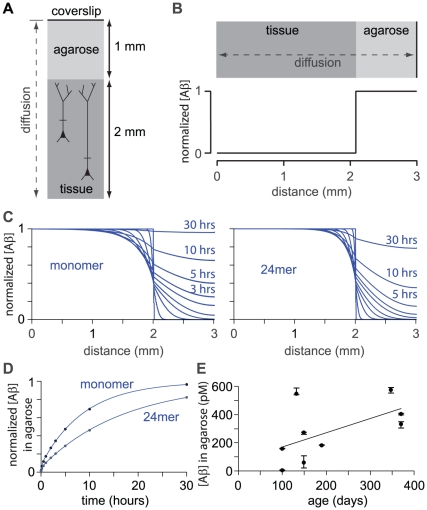
Diffusible extracellular Aβ concentration *in vivo*. (**A**) Schematic illustration of model #2, which simulates diffusion of Aβ in the intact mouse brain and overlying agarose. The model included 2mm of brain tissue and 1 mm of agarose sealed with a coverslip. We calculated diffusion in the vertical axis (dashed line). (**B**) Initial concentration of Aβ as a function of location. The tissue-agarose boundary is at 2 mm. Initial concentration in the tissue is 1, with the result that Aβ concentration is normalized to the initial concentration in brain tissue. (**C**) Concentrations of monomer and 24mer (plot as a function of location) at 1 second, 1, 10 and 30 minutes, 1, 2, 3, 5, 10 and 30 hours. Half-life of Aβ was 1.28 hours. Black lines highlight concentrations at 1 hour and 10 hours. (**D**) Mean concentration of Aβ in the agarose, plot against time, for monomer and 24mer. After 30 hours monomer and 24mer are 96.5% and 82.2%, respectively, of the initial tissue concentration. Solid lines are double exponential fits to the results. Both curves are dominated by one exponential term with time constants of 8.9 hours for the monomer and 17 hours for the 24mer. (**E**) Tissue Aβ concentration *in vivo*, calculated from measurements of Aβ concentration in agarose. Each data point represents the mean and range from one mouse (n = 9 mice; 3–4 measurements per mouse). The line is a linear fit.

First the numerical model was employed to estimate the duration for which agarose needs to be in contact with the brain for the concentration of Aβ in the agarose to reach steady-state. The model (#2) simulated diffusion from the brain into a 1 mm–thick layer of agarose (figure 5A). As in the previous model, the tissue Aβ concentration was initially set to 1 and the concentration in agarose to 0, thereby normalizing Aβ concentrations to the initial tissue concentration (figure 5B). In the model, the Aβ concentration in the superficial millimeter of brain tissue declined rapidly as Aβ was lost by diffusion into the agarose (figure 5C).

The model predicts that after 30 hours, the concentrations of Aβ in the agarose would be 97% and 82% of the initial Aβ concentration in the brain for monomer and 24mer, respectively (figure 5D). In experiments, agarose was therefore placed in contact with the brain surface for ∼60 hours ensure that the Aβ concentration in the agarose was an accurate reflection of the concentration in the brain. The concentration of Aβ_1–42_ in the agarose, and therefore of extracellular soluble Aβ_1–42_ in the brain, increased with age (figure 5E). Mean concentrations were derived from a regression line and were 160 pM at 3 months, 250 pM at 6 months and 425 pM at 12 months of age (figure 5E). These are the first measurements of the concentration of soluble Aβ in the extracellular space in CRND8 mice. The results indicate that the concentration of soluble Aβ_1–42_ in the extracellular space increases with age, much like the concentrations of Aβ_1–42_ in brain homogenates and in plasma (Chishti *et al.*, 2001; Phinney *et al.*, 2003; Hyde *et al.*, 2005).

### Release of Aβ_1–42_ from slices is greater than expected from the model

Next I measured the amount of Aβ_1–42_ released from acute brain slices from CRND8 mice. In physiology experiments, slices are typically cut from the brain and allowed to recover from the slicing process for at least 30 minutes, often several hours, before further use. To mimic a typical slice physiology experiment, after preparation, slices were placed in a large-volume holding chamber for 15–120 minutes (hereafter referred to as the recovery period). Slices were then transferred to a smaller chamber containing 250 µl ACSF and after 30 minutes the concentration of Aβ_1–42_ in the ACSF was measured using an ELISA kit.

**Figure 6:**
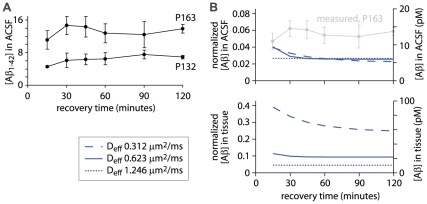
Aβ release from slices after a recovery period. (**A**) Measurements of Aβ_1–42_ concentration in ACSF as a function of recovery time for postnatal day 132 and 163 mice (P132 and P163). Slices were maintained in a large volume of ACSF for 15 to 120 minutes (x-axis) then in 250 µl of ACSF for 30 minutes. Measurements were made from three Tg+ CRND8 mice of each age. Each data point represents mean ± SEM. (**B**) Expected normalized Aβ concentrations in the ACSF and tissue, assuming a half-life for Aβ of 1.28 hours. For simplicity, tissue concentrations were averaged throughout the thickness of the slice. Results were derived from model #3, which was designed to mimic the experiment shown in panel A. The model was run with three different effective diffusion coefficients for Aβ. For comparison with the measured Aβ_1–42_ concentrations in panel A, normalized Aβ concentrations were converted into absolute concentrations by multiplying by 234 pM, which is the concentration of Aβ_1–42_ calculated from the in vivo measurements (figure 5E). The absolute concentrations therefore represent the concentrations of Aβ_1–42_ expected in P163 mice and are shown using the right hand axis. The corresponding measurements (from panel A) are overlaid in grey.

The concentration of Aβ_1–42_ in the ACSF was between 4 and 8 pM for slices from 4.5 month-old (P132) mice (figure 6A) and, as expected, the amount of Aβ_1–42_ released from slices from older mice was greater, at 11–15 pM for 5.5 month-old (P163) mice (figure 6A). The duration of the recovery period had little effect on the amount of Aβ_1–42_ released during the measurement period (figure 6A). This weak effect of the recovery period on the Aβ_1–42_ concentration in the ACSF is expected from the model, in which the Aβ concentration in the slice declines rapidly towards a steady-state concentration, in around 30 minutes. The steady-state concentrations of Aβ_1–42_ in ACSF (mean ± SEM of concentrations after 60, 90 and 120 minute recovery periods) were 7.0±0.4 pM for P132 mice and 13.0±1.1 pM for P163 mice. These concentrations are 3.5% and 5.6%, respectively, of the concentrations *in vivo* at these ages (calculated from the regression line in figure 5E), yielding a mean normalized concentration of Aβ_1–42_ in ACSF bathing slices of 0.045.

The measured concentrations of Aβ_1–42_ in the ACSF are greater than expected from the model. The model (#3) predicts that the normalized concentration of Aβ_1–42_ in the ACSF with a 60–120 minute recovery period will be 0.026 for the monomer (figure 6B) and 0.025 for the 24mer (not shown). Aβ_1–42_ *in vivo* at P132 and P163 were 203 and 234 pM. Hence the expected concentrations of Aβ_1–42_ in ACSF are 5.2 and 6.0 pM for P132 and P163 mice (figure 6B) and more Aβ_1–42_ is released into the ACSF than expected.

Could the rate of diffusion of endogenous Aβ_1–42_ be greater than expected and might this result in a greater concentration of Aβ_1–42_ in the ACSF than predicted by the model? In the model I examined the effect of changing the effective diffusion coefficient on the concentrations of Aβ in the ACSF and tissue. Both doubling and halving the effective diffusion coefficient affected the tissue concentration of Aβ, but neither doubling nor halving the effective diffusion coefficient had substantial effects on the expected concentration of Aβ_1–42_ in the ACSF (figure 6B and C). Similar effects were observed for both monomer (figure 6B and C) and 24mer (not shown). Hence even large changes in the rate of diffusion do not increase the expected concentration of Aβ_1–42_ in the ACSF. An erroneous rate of diffusion of Aβ in the model is therefore unlikely to account for the difference between expected and measured concentrations of Aβ_1–42_ in the ACSF.

### Half-life of Aβ_1–42_ in acute slices from CRND8 mice

In the model we initially used a half-life for Aβ of 1.28 hours. If the half-life of Aβ_1–42_ in acute slices is shorter than 1.28 hours, reflecting a greater rate of synthesis and/or release of Aβ_1–42_, the rate of release of Aβ_1–42_ from the slice might be elevated and the concentration of Aβ_1–42_ in the ACSF increased. This might explain the difference between expected and measured concentrations of Aβ_1–42_ in the ACSF.

**Figure 7:**
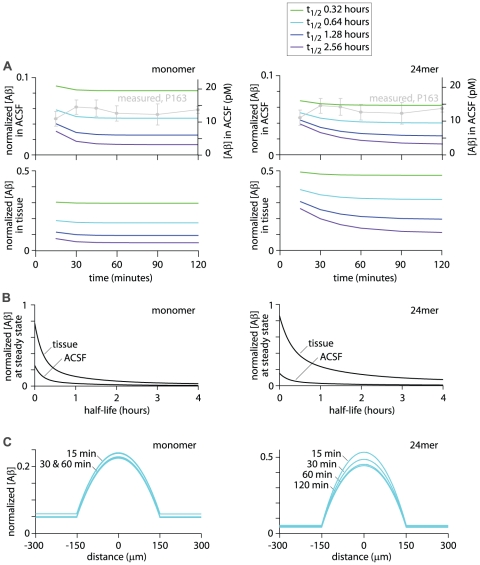
Effect of half-life on expected concentrations of Aβ in ACSF and tissue. (**A**) Predicted normalized ACSF and tissue Aβ concentrations from model #3, corresponding to the experiments in figure 6A. Normalized and absolute concentrations of Aβ are plot as a function of the recovery time. As in figure 6, absolute concentrations are calculated for P163 mice and the measured ACSF concentrations at P163 (from figure 6A) are overlaid in grey. Tissue Aβ concentrations are averages of the Aβ concentration throughout the thickness of the slice. The model was run with four half-lives of Aβ. (**B**) Steady-state normalized concentrations of Aβ in ACSF and tissue as a function of half-life of Aβ. Tissue Aβ concentration is an average of the Aβ concentration throughout the thickness of the slice. Steady-state values were taken from the concentrations at 120 minutes recovery time, which approximates the true steady-state concentrations. (**C**) Predicted normalized concentrations of Aβ as a function of location in the slice with several different recovery periods and a half-life of Aβ of 0.64 hours. Concentrations are plot as a function of location with the center of the slice at x = 0 and the edges of the slice at x = −150 and +150. Curves are shown for 3 or 4 pre-wash times (15, 30, 60 and 120 minutes).

Reducing the half-life of Aβ in the model increased the concentration of Aβ released into the ACSF (figure 7A) with the expected concentration of Aβ in the ACSF increasing approximately exponentially with decreasing half-life (figure 7B). The expected Aβ concentrations were calculated separately for monomer and 24mer and comparison with the measured Aβ_1–42_ concentrations revealed that the measured concentrations were similar to expected concentrations when the half-life was approximately 0.64 hours (figure 7A).

By adjusting the half-life of Aβ used in the model, the expected concentration in the ACSF can be matched to the measured ACSF concentration of Aβ_1–42_. From the measurements in brain slices (figure 6A) the mean normalized concentration of Aβ_1–42_ in ACSF was 0.045 (calculated above). In the model, a steady-state normalized ACSF Aβ concentration of 0.045 occurs at half-lives of 0.67 and 0.52 hours for monomer and 24mer, respectively. Hence the model best fits the measured concentrations of Aβ_1–42_ when the half-life of Aβ in the model is 0.52–0.67 hours.

### The steady-state Aβ concentration in a slice is ∼20% of the concentration *in vivo*

From the model and measurements, it is possible to estimate the tissue concentrations of Aβ. For simplicity, in figure 7A and B tissue Aβ concentration is displayed as an average of the Aβ concentration throughout the thickness of the slice. Tissue concentrations of Aβ are consistently greater than ACSF concentrations and, like ACSF concentrations, increase approximately exponentially with decreasing half-life. With a half-life of 0.64 hours, the normalized tissue concentrations of Aβ (averaged throughout the depth of the slice) were 0.16 and 0.36 for monomer and 24mer, respectively (figure 7C). If small oligomers predominate in CRND8 mice, the mean tissue concentration of Aβ at steady state in an acute slice is likely to be towards the lower end of this range, around 20% of the *in vivo* concentration.

In slices, a concentration gradient of Aβ will presumably exist, with relatively high concentrations of Aβ species deep in the slice and lower concentrations towards the edges of the slice. From the model we calculated these concentration gradients for both monomer and 24mer, using a half-life of 0.64 hours (figure 7D). The highest concentration of Aβ will be at the center of the slice. The concentration of monomer at the center of the slice was 23% of the initial concentration. Hence physiology experiments will typically report the properties of cells and synapses in relatively low concentrations of extracellular soluble Aβ. This problem may be particularly acute with whole-cell recordings, which are typically obtained from somata approximately 50 µm from the cut surface of the slice. 50 µm from the edge of the slice, the concentration of monomer was 15% of the initial concentration. For 24mer, the concentration at the center of the slice was 45% and 50 µm from the edge of the slice was 28% of the initial concentration. Hence the concentrations of soluble Aβ throughout the slice are low and in the superficial regions of the slice, where most whole-cell electrophysiological recordings are obtained, concentrations are likely to be extremely low: probably 10–20% of the concentration of Aβ *in vivo*.

### Kinetics of the changes in Aβ concentration after slice preparation

Having determined that most of the extracellular soluble Aβ is eventually lost from acute brain slices, I next investigated the rate at which the Aβ concentration approaches steady-state after slice preparation.

**Figure 8:**
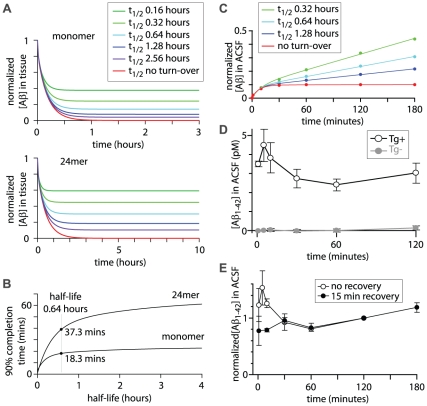
Kinetics of Aβ concentration after slice preparation. (**A**) Normalized concentrations of Aβ monomer and 24mer as a function of time after slice preparation for several different half-lives of Aβ, calculated using model #1. These are mean tissue concentrations, derived by averaging the Aβ concentrations throughout the slice. Lines are double exponential fits. (**B**) 90% completion time (time required for 90% of the decline in concentration from the initial concentration to the steady-state concentration), plot as a function of the half-life of Aβ. (**C**) Normalized ACSF Aβ monomer concentrations predicted with several different half-lives for Aβ (model #4). Each line of best fit is the sum of an exponential and a linear component. (**D**) Measurements of Aβ concentration in ACSF as a function of time. Measurements were made from three 4 month-old Tg+ CRND8 mice (open symbols) and three 5 month-old Tg− (wild-type) CRND8 mice (grey symbols). Each data point represents mean ± SEM. (**E**) Comparison of Aβ accumulation in ACSF with and without a recovery step. Measurements after a 15 minute recovery (solid symbols) were made from three 5 month-old Tg+ CRND8 mice. Mean ACSF Aβ concentration with the recovery period was 11.3±2.5 pM after 2 hours. For comparison with the results from panel A (open symbols), ACSF Aβ concentrations were normalized to that at 2 hours.

I first used the model to calculate the expected time course of Aβ in the tissue. The tissue concentrations (averaged throughout the depth of the slice) of monomer and 24mer both declined rapidly towards steady-state concentrations (figure 8A). The time taken to reach a steady-state concentration of Aβ in the tissue was expressed as the 90% completion time, defined as the time taken for the concentration of Aβ to decline by 90% of the difference between the initial concentration and the steady-state concentration. The steady-state concentrations decreased and the 90% completion time increased with increasing half-life (figure 8A and B). As half-life tended towards infinity (no turn-over of Aβ), the 90% completion time tended towards asymptotes of 23.3 minutes for the monomer and 66 minutes for the 24mer. Hence the model predicts that the 90% completion times in slices (in which there is turn-over of Aβ) are less than 23.3 minutes for monomer and 66 minutes for 24mer. For a half-life of 0.64 hours, the predicted completion times were 18.3 minutes for monomer and 37.3 minutes for 24mer.

To measure the kinetics of the changes in Aβ concentration after slice preparation, each slice was placed in a small-volume chamber, containing 250 µl of ACSF, immediately after preparation. The concentration of Aβ_1–42_ in the ACSF was measured after 1 to 180 minutes in the well. The model (#4) predicts an initial rapid increase in the concentrations of monomer (figure 8C) and 24mer (not shown) in the ACSF. This initial phase lasts less than 30 minutes (figure 8C). Thereafter the ACSF concentration continues to rise approximately linearly, with the gradient increasing with decreasing half-life of Aβ (figure 8C).

In contrast to the predictions of the model, the measured Aβ_1–42_ concentration decreased during the first hour after slice preparation. ACSF Aβ_1–42_ concentration declined by approximately 40%, from a peak of 4.5±0.85 pM at 5 minutes to 2.4±0.31 pM at 1 hour (P<0.05, paired t-test; figure 8B). Thereafter the ACSF Aβ_1–42_ concentration stabilized and possibly increased slightly during the next hour (to 3.0±0.5 pM at 2 hours; not significantly different from concentration at 1 hour).

This decline in ACSF Aβ_1–42_ concentration with time was unexpected. I considered whether this might be a methodological artifact, such as binding of Aβ to the plastic walls of the 24-well plate. To test for this, measurements were repeated with a recovery period in which each slice was placed in a large volume of ACSF for 15 minutes before being transferred to the 24-well plate. A methodological artifact, such as binding to plastic, would be unaffected by the recovery period and the decline would therefore persist. In contrast, much of the Aβ_1–42_ from an initial elevation in Aβ_1–42_ would exit the slice within <30 minutes, reducing the Aβ_1–42_ concentration in the first few wells and suppressing the decline in Aβ_1–42_.

Normalized ACSF concentrations of Aβ_1–42_ were initially (t≤10 minutes) lower with than without the recovery step (figure 8E) and the decline in ACSF Aβ_1–42_ concentration with time was eliminated. For both conditions, the Aβ_1–42_ concentration increased gradually after 1 hour (figure 8E). Hence a 15 minute recovery period reduced the initial elevation and subsequent decline in Aβ_1–42_ concentration, indicating that the changes in Aβ_1–42_ concentration are not a methodological artifact. Hence Aβ_1–42_ was removed from the ACSF during the first hour after slice preparation. Immediately after slice preparation the extracellular diffusible Aβ_1–42_ concentration therefore follows a complex kinetic scheme: it initially increases, likely as a result of release of Aβ_1–42_ during slice preparation, and then declines over the first 30–60 minutes before reaching a steady-state condition.

Why does the concentration of Aβ_1–42_ in the ACSF decline with time? Presumably Aβ_1–42_ is taken-up by the tissue or catabolized via one of the many pathways for enzymatic degradation of Aβ_1–42_ [33]. Uptake and metabolism of Aβ are incorporated into the model through the half-life calculations. In the model, as Aβ concentration in the tissue declines, the balance between Aβ release/synthesis and Aβ uptake/catabolism tips towards net release/synthesis. This imbalance, along with diffusion of Aβ, results in the increase in ACSF Aβ concentration with time (figure 8C). The measured decline in Aβ_1–42_ concentration (figure 8D) suggests that uptake/catabolism and release/synthesis are unbalanced in the other direction, resulting in net uptake/catabolism. Such an unbalance might occur if the concentration of Aβ is elevated. Slicing brain tissue inevitably results in the death of neurons, particularly near the cut surfaces of the slice, and dying cells may release Aβ. In addition, slice preparation may elevate synaptic activity, which can release Aβ from synaptic terminals [20], [34]. Aβ concentration may therefore be elevated immediately following slice preparation.

**Figure 9:**
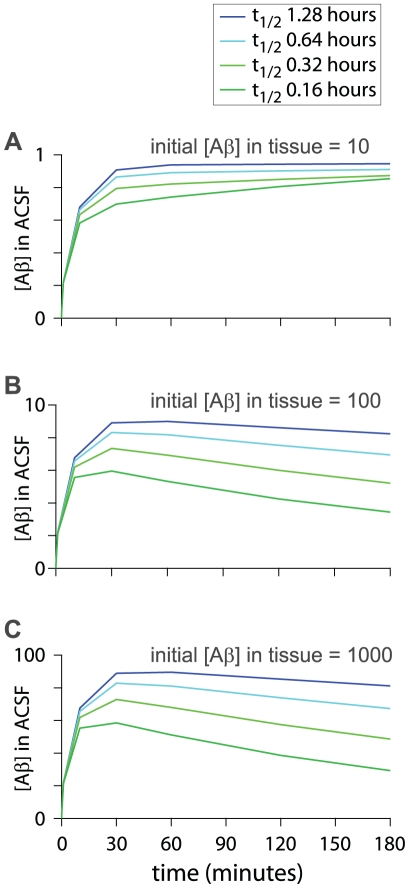
Effects of initial Aβ concentration and half-life of Aβ on the ACSF Aβ concentration. Predicted ACSF Aβ concentrations as a function of time in model #4. The model was run with four half-lives of Aβ for three elevated initial Aβ concentrations in the tissue: [Aβ] = 10 (A); [Aβ] = 100 (B); [Aβ] = 1000 (C).

Elevating the initial Aβ concentration in the model resulted in a decline in ACSF Aβ concentration with time (figure 9). The decline became faster and more pronounced as the initial Aβ concentration was increased and the half-life for Aβ was decreased (figure 9), but the decline predicted by the model was slow, presumably because the slice occupies only a small proportion (10%) of the volume of the chamber. Elevating the initial Aβ concentration in the model, even by several orders of magnitude, failed to reproduce the observed rapid decline in ACSF Aβ_1–42_ concentration. Hence release of Aβ during slice preparation is unlikely to entirely account for the decline in ACSF Aβ concentration during the first hour and additional factors, that are not incorporated in the model, are likely responsible for the decline in Aβ_1–42_ concentration after slice preparation.

### Kinetics of exogenous Aβ accumulation in slices

**Figure 10:**
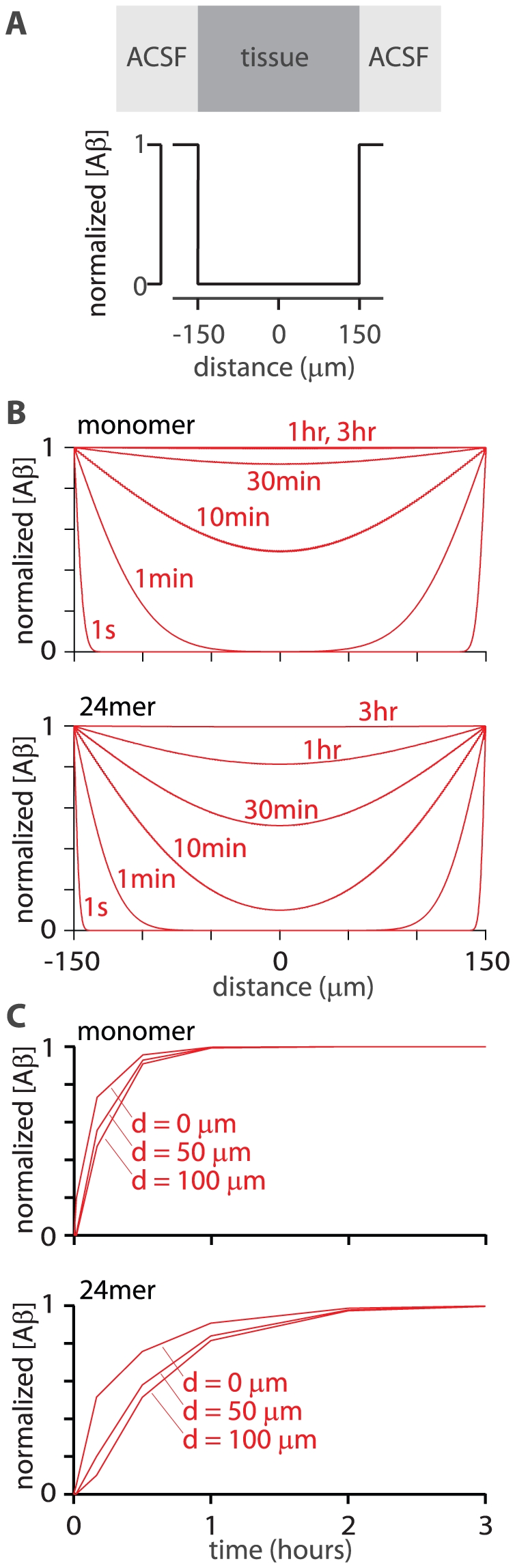
Diffusion of exogenous Aβ into acute slices. (**A**) Initial concentrations of Aβ. Tissue-ACSF boundaries are at distances of +150 and −150 µm. Initial concentration in the tissue is zero and in the ACSF is 1, with the result that all concentrations in the model are normalized to the initial concentration in the ACSF. (**B**) Concentrations of monomer and 24mer at all locations in the brain slice (plot as a function of distance from the center of the brain slice) at 1 second, 1 minute, 10 minutes, 30 minutes, 1 hour and 3 hours (**C**) Concentrations of monomer and 24mer as a function of time at three different locations within the brain slice. Distances (0, 50, 100 µm) are with respect to the center of the slice.

Finally I used the model to calculate the rate at which Aβ enters a slice when the slice is bathed in ACSF containing exogenous Aβ. In this model (#5) the initial concentration of Aβ in the slice was set to zero and the concentration in ACSF to one (figure 10A). The concentration of Aβ in the slice approached the concentration in the ACSF within 30 minutes (monomer) to 1 hour (24mer) throughout the depth of the slice (figure 10B and 10C). Hence diffusion of soluble Aβ species in brain tissue not only results in the rapid loss of endogenous extracellular Aβ from slices, but also a rapid rise in Aβ concentration within the slice during the application of exogenous Aβ.

## Discussion

I have measured extracellular Aβ_1–42_ concentrations *in vivo* and in acute brain slices from CRND8 mice. The extracellular concentrations of Aβ species have been measured previously in the intact mouse brain by microdialysis and ELISA [30], but the size of microdialysis probes precludes their use in acute slices, which are only 200–400 µm thick. Instead I determined the extracellular concentration of Aβ_1–42_ in acute brain slices indirectly, measuring the concentration of Aβ_1–42_ released and using a numerical model to calculate, from these measurements, the extracellular concentration of diffusible Aβ_1–42_ in the tissue.

My principal conclusion is that the concentration of Aβ_1–42_ in acute slices is reduced by the loss, by diffusion, of most of the soluble extracellular Aβ_1–42_. The steady-state concentration of Aβ_1–42_ in the slice was dependent on the parameters used in the model, but the calculated Aβ_1–42_ concentration was greatly reduced across a wide range of model parameters. Near the surfaces of the slice, the calculated steady-state Aβ_1–42_ concentration was less than 20% of the concentration *in vivo* and even deep within the slice the concentration was only ∼20–30% of the *in vivo* concentration.

### Heterogeneous distribution of Aβ and plaques in CRND8 mice

CRND8 mice carry two APP mutations (KM670/671NL ‘Swedish’ and V717F ‘Indiana’) and elevated levels of Aβ in hippocampus and through much of the neocortex from a young age [35]. CRND8s develop many of the histological and cognitive deficits of patients with AD, including amyloid plaques in hippocampus and neocortex [16], [35]–[37]. CRND8 mice do not carry mutations in other genes or exhibit tau hyperphosphorylation or neurofibrillary tangles and are therefore an excellent model in which to study the effects of Aβ.

In my models I treated the slice as homogenous, but for slices from CRND8 mice, hippocampus and neocortex are likely to be the main sources of Aβ released into the ACSF. Hippocampus and neocortex together account for ∼50% of the coronal slices used in my experiments. Hence if little or no Aβ is released from other regions, the amount of Aβ released per unit volume of hippocampal and neocortical tissue might be higher than calculated in the results. The loss of Aβ from slices may therefore occur more rapidly than suggested by my simplified models and my calculations likely provide a conservative estimate of the rate of Aβ loss from acute slices.

Amyloid plaques first appear in the hippocampus and neocortex of CRND8 mice at 2–3 months of age and the plaque load increases steadily thereafter. Most of the mice used here were 2–6 months old, ages at which the plaque load is light. Whether plaques affect diffusion of soluble Aβ species is unclear, but it is possible that the increasing plaque load with age would impede diffusion of Aβ and retard loss of Aβ from slices. However, given the relatively small percentage of the total volume of hippocampus and neocortex occupied by plaques, even in old mice, it seems unlikely that plaques would substantially alter diffusive loss of Aβ from acute slices.

One interesting question is whether changes in the diffusion of Aβ in aging tissue contribute to plaque deposition. My results do not address this issue and this therefore remains an intriguing possibility that requires further investigation.

### Accuracy of the model and the half-life of Aβ

The model benefits from being reasonably simple, with most parameters available from publications. The initial model, which used values or reasonable estimates from the literature, predicted concentrations of Aβ that are close to the measured concentrations, indicating that the model is a good starting point from which to consider loss of Aβ from acute slices. Nevertheless, measured steady-state ACSF concentrations of Aβ_1–42_ were approximately 2-fold greater than the concentration predicted by the model. What might account for this difference?

The principal variables in the model are the rate of diffusion and the half-life of Aβ. An error in the rate of diffusion is unlikely to account for the difference between predicted and measured Aβ concentrations. Accurate values for the diffusion coefficient of Aβ and for the tortuosity factor, which accounts for restrictions on diffusion in the extracellular space, are available in the literature [25]–[28] and diffusion of fluorescent Aβ_1–42_ is consistent with these calculated rates of diffusion. No adjustment was made to account for binding of Aβ oligomers in the extracellular space, to excitatory synapses for example [38], but binding would slow, not speed diffusion of Aβ species through the extracellular space and the absence of binding in the model would therefore not result in an underestimate of Aβ release. Furthermore, changing the rate of diffusion in the model had little effect on the amount of Aβ released from the slice. Hence changes in diffusion do not explain the difference between initial model and measurements.

The half-life of Aβ in the model is a more likely source of inaccuracy as the half-lives of Aβ species are less clear from the literature. There are few published half-life measurements from APP overexpressing mice. Measured half-lives range from 0.7 and 1.7 hours or less for Aβ_1–40_ and Aβ_1–42_, respectively (measured by postmortem extraction of Aβ from brain tissue [31]) to 4 hours (measured by *in vivo* dialysis [30]). A half-life of 1.28 hours was initially used in the model and 0.52–0.67 hours was found to more accurately reproduce the measured concentration of Aβ_1–42_ released from slices. 0.64 hours was therefore used to calculate the concentrations of Aβ_1–42_ in the tissue. 0.64 hours is towards the shorter end of the range of published half-lives. However, the published half-lives were based on *in vivo* measurements. Slice preparation likely results in a temporary increase in neuronal activity, which may increase release of Aβ species, and the release of Aβ metabolizing enzymes from damaged cells. Hence the half-lives of Aβ species in slices are likely to be shorter than half-lives *in vivo*.

A third possible explanation is that the initial concentration of Aβ_1–42_ in the slice is lower than expected. The expected value was derived from the relationship between Aβ_1–42_ concentration and age *in vivo*. The *in vivo* measurements revealed enormous variability in Aβ_1–42_ concentrations between mice (figure 5E). The reason for this variability is unclear, but presumably reflects differences in APP expression and/or Aβ accumulation between individual mice. As a result, any individual mouse brain might contain far more or far less Aβ_1–42_ than an average mouse, opening the possibility that the concentrations of Aβ_1–42_ in the brains of mice used in slice experiments were higher than the mean value from *in vivo* experiments. If this were the case, a longer half-life for Aβ would be consistent with the measured release of Aβ_1–42_ from slices (the grey line would move downwards in figure 7A). However, this would have very little effect on the steady-state concentrations of Aβ_1–42_ in slices and only a modest effect on the rate at which this steady-state is approached (figure 8A and B).

The model failed to explain the rapid decrease in Aβ_1–42_ concentration in ACSF in the first hour after slice preparation (Figures 6C and 7). This failure might result from the manner in which half-life was implemented in the model, as a constant rate of Aβ production and a constant percentage rate of elimination. In slices, declining Aβ concentration might stimulate Aβ synthesis or release, resulting in changes in the half-lives of Aβ species through time. A transient elevation in half-life could explain the rapid decline in ACSF Aβ_1–42_ concentration immediately after slice preparation and might occur if Aβ metabolizing enzymes are released from damaged cells, transiently increasing the rate of Aβ clearance from the slice.

Other simplifications in the model could also contribute to the difference between expected and measured Aβ concentrations. For example, in the model the tissue is considered homogeneous, whereas diffusion of Aβ is likely to differ between regions of the slice, between white and grey matter for example. In addition, the initial concentrations of Aβ species are unlikely to be homogeneous throughout the brain. In CRND8 mice, APP overexpression and Aβ accumulation are pronounced in hippocampus and neocortex [35]. These regions may be the principal source of Aβ released from the slice by diffusion, in which case loss of Aβ_1–42_ from hippocampus and neocortex would have to be particularly severe to elevate ACSF Aβ_1–42_ to the measured concentrations and the concentrations of Aβ_1–42_ remaining in hippocampus and neocortex in an acute slice would be lower than calculated here. My calculations of the percentage loss of Aβ from acute slices may therefore be conservative estimates. Even these conservative estimates indicate that the loss of Aβ from acute slices is profound.

### Effects of Aβ_1–42_ on synaptic physiology

ELISA assays have been used previously to measure Aβ concentrations in the brains of mice overexpressing APP. Previous measurements were made on homogenized tissue and therefore include both intra- and extracellular Aβ. In contrast, I measured Aβ_1–42_ that diffused from the brain into an overlying layer of agarose *in vivo*. Hence the membranes of brain cells remained intact and the measured concentration was of only extracellular diffusible Aβ_1–42_.

I concluded that the concentration of extracellular diffusible Aβ_1–42_ in CRND8 mice increases from 160 pM to 425 pM as CRND8 mice age from 3 to 6 months. The plasma concentration of Aβ_1–42_ in 6 month-old CRND8 mice is 0.7–2 nM [36], [39]. Plasma and cerebrospinal fluid concentrations of Aβ are similar [40]–[42]. Hence the *in vivo* Aβ_1–42_ concentrations reported here are consistent with previous measurements from CRND8 mice.

At these concentrations Aβ affects long-term potentiation (LTP) when applied to acute hippocampal slices. At high picomolar and nanomolar concentrations Aβ oligomers inhibit LTP (700 pM [18]; 500 nM [19]; 200 nM [22]) whereas both inhibition and enhancement have been reported with 50–200 pM Aβ [22], [24]. Hence the extracellular concentrations of soluble Aβ_1–42_ in 2–6 month-old CRND8 mice, may enhance or suppress synaptic plasticity *in vivo*.

Deficits in synaptic plasticity attributable to soluble Aβ species have also been observed in acute slices from CRND8 and other APP overexpressing mice. Hence loss of soluble Aβ from slices does not eliminate all the effects of soluble Aβ species on synaptic transmission, perhaps because these effects of Aβ are irreversible or slowly reversible. However, the effects of extracellular soluble Aβ_1–42_ can occur within only a few minutes of exposure to Aβ_1–42_. If some of the effects of Aβ are also rapidly reversible, these effects may be lost or moderated after slice preparation. For example, in acute slices from 2–6 month-old CRND8 mice the effect on LTP may be lost as the concentration of soluble extracellular Aβ_1–42_ declines from 300–500 pM *in vivo* to 100 pM or less in slices. As the mice age, and the soluble extracellular Aβ_1–42_ increases, the effects of loss of Aβ may change. The rate at which loss of Aβ alters synaptic physiology in slices is unclear. Loss of Aβ_1–42_ from acute slices is rapid, but Aβ molecules bind to plasma membranes [38]. Hence Aβ might remain bound to target molecules despite the falling extracellular concentrations of diffusible Aβ species.

I also estimated the rate of entry of exogenous Aβ, applied in the ACSF. The model predicts that exogenous Aβ enters the slice quickly, reaching a steady-state concentration equivalent to the concentration in ACSF in 30 minutes to an hour. Unsurprisingly, this time course is similar to that for loss of endogenous Aβ. Turn-over of Aβ was not considered in the model of exogenous Aβ entry. Some authors have applied exogenous Aβ at extremely high concentrations, far higher than endogenous concentrations of Aβ (e.g. [43]). At these concentrations the relevant metabolic pathways may be unable to significantly affect Aβ concentration and it is therefore unclear how to model turn-over in these cases. Fortunately in my models the effect of turn-over on the rate of change of concentration is relatively weak (see figure 8 A and B, for example). My results suggest that effects of exogenously applied Aβ observed in less than 30–60 minutes are likely to result from lower concentrations of Aβ than those in the perfusing ACSF and that this Aβ must be acting extremely quickly. For example, effects of exogenous Aβ on synaptic plasticity have been reported following application of only 50–200 pM exogenous Aβ for 20 minutes (e.g. [22], [24]). My results suggest that such effects must result from a site of action which is exclusively on the surface of the slice or from an extremely rapid action of lower concentrations of soluble Aβ species than are present in the extracellular space in many APP mouse lines.

In summary, here I have shown that the concentration of soluble Aβ_1–42_ in the extracellular space declines during the first hour after preparation of acute brain slices from CRND8 mice. Electrophysiological recordings in acute slices are usually obtained at least 30 minutes and often several hours after slice preparation. Hence recordings in acute slices from APP overexpressing mice are obtained when extracellular Aβ is at a steady-state concentration, which is far lower than the concentration of Aβ *in vivo*. In acute slices prepared from CRND8 mice or other APP overexpressing mouse lines, diffusive loss of Aβ from the tissue after slice preparation may eliminate or moderate some of the effects of Aβ, greatly complicating interpretation of physiology experiments aimed at elucidating the effects of Aβ.

## Supporting Information

Code S1 Igor packed experiment, containing code for model #1.
(PXP)
